# Native Fungi as a Nature-Based Solution to Mitigate Toxic Metal(loid) Accumulation in Rice

**DOI:** 10.3390/microorganisms13071667

**Published:** 2025-07-16

**Authors:** Laura Canonica, Michele Pesenti, Fabrizio Araniti, Jens Laurids Sørensen, Jens Muff, Grazia Cecchi, Simone Di Piazza, Fabio Francesco Nocito, Mirca Zotti

**Affiliations:** 1 Laboratory of Mycology, Department of Earth, Environment and Life Science, University of Genoa, Corso Europa 26, 16132 Genoa, Italy; laura.canonica@edu.unige.it (L.C.); grazia.cecchi@edu.unige.it (G.C.); simone.dipiazza@unige.it (S.D.P.); 2 Dipartimento di Scienze Agrarie e Ambientali—Produzione, Territorio, Agroenergia, Università degli Studi di Milano, Via Celoria 2, 20133 Milano, Italy; michele.pesenti@unimi.it (M.P.); fabrizio.araniti@unimi.it (F.A.); fabio.nocito@unimi.it (F.F.N.); 3 Department of Chemistry and Bioscience, Aalborg University, Niels Bohrs Vej 8A, 6700 Esbjerg, Denmark; jls@bio.aau.dk (J.L.S.); jm@bio.aau.dk (J.M.)

**Keywords:** arsenic, bioinoculants, cadmium, chromium, contamination, copper, fungal traits, metabolites, sustainable agriculture

## Abstract

Heavy metal contamination in paddy fields poses serious risks to food safety and crop productivity. This study evaluated the potential of native soil fungi as bioinoculants to reduce metal uptake in rice cultivated under contaminated conditions. Eight fungal strains—four indigenous and four allochthonous—were selected based on their plant growth-promoting traits, including siderophore production and phosphate solubilization. Additional metabolic analysis confirmed the production of bioactive secondary metabolites. In a greenhouse experiment, three rice cultivars were grown under permanent flooding (PF) and alternate wetting and drying (AWD) in soil enriched with arsenic, cadmium, chromium, and copper. Inoculation with indigenous fungi under AWD significantly reduced the arsenic accumulation in rice shoots by up to 75%. While AWD increased cadmium uptake across all cultivars, fungal inoculation led to a moderate reduction in cadmium accumulation—ranging from 15% to 25%—in some varieties. These effects were not observed under PF conditions. The results demonstrate the potential of native fungi as a nature-based solution to mitigate heavy metal stress in rice cultivation, supporting both environmental remediation and sustainable agriculture.

## 1. Introduction

Rice is one of the most important staple foods globally, consumed by approximately 50% of the world’s population [1,2,3]. The growing presence of toxic non-essential metals and metalloids (e.g., cadmium and arsenic) in paddy fields threatens both rice food safety and yield [4,5,6]. These elements, originating from anthropogenic activities or natural sources, are inherently toxic and tend to accumulate in the environment due to their non-biodegradable nature [7,8,9,10]. The accumulation of toxic metal(loids) in these soils affects fertility, reduces crop yields, and often results in the production of metal-contaminated food [11,12]. Consequently, several reference guidelines are available to identify critical contamination thresholds in soils. For example, the Finnish guidelines offer threshold values that are often referenced in the context of European environmental assessments [13,14]. Moreover, high concentrations of metal(loids) interfere with plant metabolism and disrupt plant functions by interfering with protein structures, enzyme activity, and membrane integrity [15,16]. Finally, toxic levels of metal(loids) promote the overproduction of reactive oxygen species and cytotoxic compounds, leading to oxidative stress. This imbalance damages lipids, proteins, and nucleic acids and ultimately triggers programmed cell death [15,17,18,19].

In the presence of this abiotic stress, plants exhibit an increased vulnerability to phytopathogen attack, which poses a serious risk to crop yield [20,21,22]. In light of these considerations, the development of sustainable management strategies for contaminated agricultural soils is becoming increasingly urgent, particularly because of global population growth and rising food demands [23]. Current approaches, such as the application of amendments, organic fertilizers, or biochar, focus on modifying key parameters that influence the bioavailability of metals, e.g., the cation exchange capacity [1,24,25]. Nonetheless, the implementation of specific methodologies may impact crop-associated microbiota [23,26]. The community of root-associated microorganisms plays a biostimulatory, protective, and filtering role in the surrounding soil [27,28]. In the context of polluted soils, the presence of metal-resistant systems of fungi (and of bacteria) has been demonstrated to reduce the bioavailability of metals to plants. It is evident that these microorganisms have been developed multiple strategies to mitigate metal-induced stress. These strategies encompass a range of processes, including enzymatic detoxification, active or passive uptake, adsorption on cell surfaces, precipitation on cell surfaces, exclusion by permeability barriers, and the action of efflux pumps [29,30,31]. Interest in plant growth-promoting bacteria and fungi (PGPB and PGPF) has increased significantly in recent years due to their ability to synthesize and release functional metabolites in the rhizosphere. These include the synthesis of phytohormones such as indole-3-acetic acid (IAA), as well as the production of siderophores. Additionally, they facilitate the solubilization of phosphate and the release of various bioactive compounds, such as cellulase and chitinase, which play crucial roles in nutrient cycling and the decomposition of organic matter. Together, these microbial activities enhance nutrient availability and promote the health of both plants and soils [32,33,34,35]. In addition to the involvement of bacteria in stress tolerance, fungi have also been implicated in the adaptation of plants to various extreme habitats through different tolerance mechanisms [6,36,37]. As reported in several reviews, numerous studies have explored the use of fungal organisms in combination with plants of agricultural interest [33,34,38]. Regarding rice cultivation, most research has focused on the effect of fungi on plant resistance to abiotic stress related to drought, heat, and salinity conditions [39]. Conversely, studies on rice plants under metal contamination were more limited in scope, with a primary focus on the application of arbuscular mycorrhizal fungi [40,41]. This research aimed to investigate the effects of microfungi as bioinoculants on the accumulation of heavy metals in rice plants. First, the metabolic traits of selected fungal strains isolated from paddy field soils were investigated. Then, the impact of inocula, comprising both indigenous and allochthonous fungi, was assessed on three rice varieties grown under two distinct cultivation regimes in soil with elevated heavy metal concentrations. This work proposes an innovative approach based on the development of bioinoculants composed of autochthonous fungal strains isolated from polluted soils. The strains were specifically selected for their distinctive metabolic traits, aimed at mitigating ecotoxicant-induced stress and enhancing the bioavailability of key nutrients, such as phosphorus.

## 2. Materials and Methods

### 2.1. Isolation and Identification of Fungal Strains

The fungal strains employed were previously isolated from paddy field soils and cryopreserved at the ColD UNIGE JRU MIRRI-IT collection (Laboratory of Mycology, University of Genoa). The identification of the fungi was conducted through a morphological and molecular identification approach. In the latter, genomic DNA was extracted from 100 mg of fresh fungal culture, according to the Doyle and Doyle method [42]. PCR amplifications were performed using the internal transcribed spacer (ITS) region as the primary barcodes, along with the genus specific markers beta-tubulin (BT), calmodulin (CMD), and ribosomal large subunit (LSU) as secondary barcodes [43,44,45,46]. The sequences were deposited in GenBank with the following accession numbers: OQ248241.1, from PV474609 to PV474611, from PV480867 to PV480872, and from PV751089 to PV751092.

### 2.2. Fungal Functional Trait Tests Set-Up

Three different assay media were prepared: Pikovskaya’s medium (PKV), Chrome-Azurol-S medium (CAS), and a Creatine Sucrose Agar medium (CREA) [34,47,48].

PKV medium was prepared using PKV agar H by dissolving 31.3 g of powder in 1000 mL of distilled water, sterilizing at 121 °C for 15 min, and then pouring the mixture into sterile Petri plates [49]. For the CAS medium, 900 mL of Malt Extract Agar base medium (MEA) was prepared, and the CAS components (Chrome Azurol S, HDTMA, and FeCl_3_·6H_2_O) were mixed, forming a deep blue solution which was gradually buffered with a 0.1 M NaOH solution to reach pH 6.8. Both the CAS solution and MEA medium were sterilized separately, then combined at 85 °C, and poured into Petri dishes to cool [50,51]. The CREA medium was prepared by mixing agar, bromocresol purple, creatine monohydrate, sucrose, trace metal, and mineral solutions, then autoclaved, and poured into Petri plates [52,53]. Point inoculation was performed using a sterile loop immersed in a suspension of fungal biomass—obtained from a 7-day pure colony grown on a 90 mm Potato Dextrose Agar (PDA) plate—and sterile deionized water. The ratio of halo (H) to colony diameter (C)—expressed as H/C—was determined based on seven-day growth measurements for each fungus and treatment.

### 2.3. Secondary Metabolites Production Assessment

Three different media were prepared: MEA, Czapek Yeast Ex-tract Agar (CYA), and Yeast Extract Sucrose (YES) [54]. The media were autoclaved at 121 °C for 15 min. Three-point inoculation was performed for each plate, and three replicates were set up for each medium. The plates were then incubated at 25 °C in the dark for 14 days. Nine agar plugs obtained from each plate were transferred into a glass tube, and 2 mL of the extraction solvent (composed of ethyl acetate, dichloromethane, and methanol in a 3:2:1 ratio with 1% formic acid) was added. The mixture was subjected to ultrasonic extraction for 45 min at room temperature (Branson 3510 Ultrasonic Cleaner; Branson Ultrasonics Corporation, Dunbury, Connecticut, USA), according to Frisvad 2012 [54]. After extraction, the solvent was transferred to a new vial and evaporated to dryness under a stream of nitrogen at 40 °C. The dried samples were dissolved by adding 600 μL of methanol, and the solution was sonicated for 10 min. The solution was then centrifuged at 10,000× *g* for 2 min (Minispin; Eppendorf, Hamburg, Germany), and the supernatant was transferred to new 2 mL screw-top vials for metabolic profile analyses. This was conducted by high-performance liquid chromatography (Hitachi Elite LaChrom HPLC; Hitachi High-Tech Corporation, Tokio, Japan) coupled to a high-resolution mass spectrometer (HRMS; Bruker compact MS ESI-Q-TOF; Bruker Daltonics GmbH & Co. KG, Bremen, Germany), as previously described [55,56]. The identification was carried out using the internal dedicated database.

### 2.4. Composition and Enrichment of Bioinoculants

Eight fungal strains were selected and distributed into two distinct inocula, each composed of four native and four allochthonous fungal strains. The strains were cultured on 120 mm MEA plates. For each fungal strain, four replicate plates were prepared, resulting in a total of 32 plates. Each plate was inoculated with 1 mL of a solution composed of seven-day-old fresh biomass from two 60 mm plates and deionized water. The plates were incubated at 24 °C in the dark for two weeks. A 300 mL suspension of each fungal strain was prepared for distribution in the soil. The suspensions, which comprised fungal biomass and sterile deionized water, were subjected to a one-hour shaking process. Thereafter, the solutions were combined in sterile 2-liter sampling bottles to constitute the two target inocula. To ensure uniformity, the solutions were homogenized. The conidia and fungal fragments in each inoculum were then quantified using a Bürker chamber and light microscopy.

### 2.5. Greenhouse Experiment Set-Up

The greenhouse experiment was set up using three different rice varieties (Cripto, Titanio, and Plus) that were grown under two distinct water regimes: permanent flooding (PF) and alternate wetting and drying (AWD). The experiment was conducted using soil from a paddy field with high levels of metal contamination.

For each combination of rice variety and cultivation regime, the two fungal inocula were applied. A control condition—rice plants without fungal inoculation—was also included for comparison. A total of 72 square pots (8 × 8 × 8 cm) were prepared: 48 contained fungal inocula, while the remaining 24 served as uninoculated controls. This methodology resulted in four replicates for each variety and cultivation regime. The pots were distributed in six separate tubs. Each pot was inoculated with 50 mL of the respective fungal suspension. Subsequently, nine seeds were sown in each pot. The trial was conducted and monitored for 15 weeks.

Following the initial germination phase, a thinning process was performed during the fourth week to retain a single well-developed plant in each pot. Consequently, the plants were subjected to the corresponding irrigation treatments. For the permanent flooding (PF) treatment, the pots were filled with water until it reached 3–4 cm above the soil surface. The water level was consistently maintained throughout the experiment. The pots containing the plants to be subjected to the alternate wetting and drying cultivation regime were kept in a dry phase until the field capacity reached 30%, at which point the flooding process was initiated. To determine the soil moisture at field capacity, undisturbed soil was first saturated with water and allowed to drain under gravity for 24 h. A soil sample was then collected, weighed, oven-dried at 105 °C for 24 h, and subsequently reweighed. The achievement of 30% field capacity during the dry phase was monitored by weighing the pots daily, allowing for the precise control of soil moisture levels before re-irrigation. The experiment was conducted in the greenhouse under constant monitoring, with a long daytime photoperiod of 16 h of light and 8 h of darkness at a temperature of 26 °C during the day and 22 °C at night. Lumatek Pro 600 W support lamps were utilized (light intensity of 300–350 µmol/m^2^/s).

At the end of the growth period, the aerial parts of the plants were harvested, oven-dried at 65 °C until constant weight, and subsequently ground into a fine powder using a laboratory agate ball mill grinder to minimize the risk of metal contamination during sample preparation. Subsamples of 100 mg of the powdered material were digested using a microwave digestion system (Anton Paar MULTIWAVE 3000; Anton Paar GmbH, Graz, Austria). Digestion was performed in Teflon vessels containing 10 mL of 65% HNO_3_, following a two-step power ramp: Step 1 at 500 W for 10 min followed by a 5 min hold and Step 2 at 1200 W for 10 min followed by a 15 min hold. After a 20 min cooling period, the digested samples were transferred into polypropylene test tubes. The digestion parameters were optimized using certified reference materials of dried plant powders with known elemental compositions.

The samples were diluted 1:20 with Milli-Q water, and the concentrations of As, Cd, Cr, and Cu were determined using inductively coupled plasma mass spectrometry (ICP-MS, Varian 820 ICP-MS). An aliquot of an internal standard solution (containing ^20^Sc, ^89^Y, and ^159^Tb at 2 mg/L) was added to both samples and calibration standards to achieve a final concentration of 20 µg/L. Potential polyatomic interferences were mitigated using a collision–reaction interface (CRI) with a hydrogen gas flow rate of 45 mL/min.

Statistical analysis was carried out using SigmaPlot for Windows version 11.0 (Systat Software, Inc.). Quantitative values are presented as the mean ± standard deviation of the mean (SD) from two experiments run in quadruplicate. ANOVA was carried out using SigmaPlot for Windows version 11.0 (Systat Software, Inc., San Jose, CA, USA). Significance values were adjusted for multiple comparisons using the Bonferroni correction. Student’s *t*-test was used to assess the significance of the observed differences between PF and AWD.

### 2.6. Vitality Tests and Chemical Analysis

Two fungal viability tests were employed during the trial to verify the presence and persistence of the strains within the inocula at weeks 4 and 12, respectively [57,58]. One gram of dried soil was serially diluted to reach the target concentration of 1:10,000, and 1 mL of the diluted solution was plated onto Malt Extract Agar media supplemented with chloramphenicol (MEA+C) and Rose Bengal (RB) media. The analysis was performed in triplicate for each type of medium and inoculum regime. The plates were incubated in the dark at 24 °C for 7 days and subsequently monitored for an additional 7 days.

## 3. Results

### 3.1. Fungal Identification and Functional Trait Tests

A total of eight fungal strains were isolated from the rice paddy soils and subsequently identified. Four of these were collected from the contaminated paddy soil that was used in the greenhouse experiment: *Aspergillus flavus* Link (028-G1), *Mortierella elongata* Linnem (028-G2), *Penicillium* sp.(029-A6), and *Penicillium chalabudae* Visagie (029-A5). The other four strains isolated from different paddy field soils were identified as follows: *Acrostalagmus luteoalbus* (Link) Zare (PAV32), W. Gams & Schroers, *Penicillium piscarium* Westling (022-I8), *Trichoderma harzianum* Rifai complex (022-D5), *Penicillium glandicola* (Oudem.) Seifert & Samson (PAV 25).

**Figure 1:**
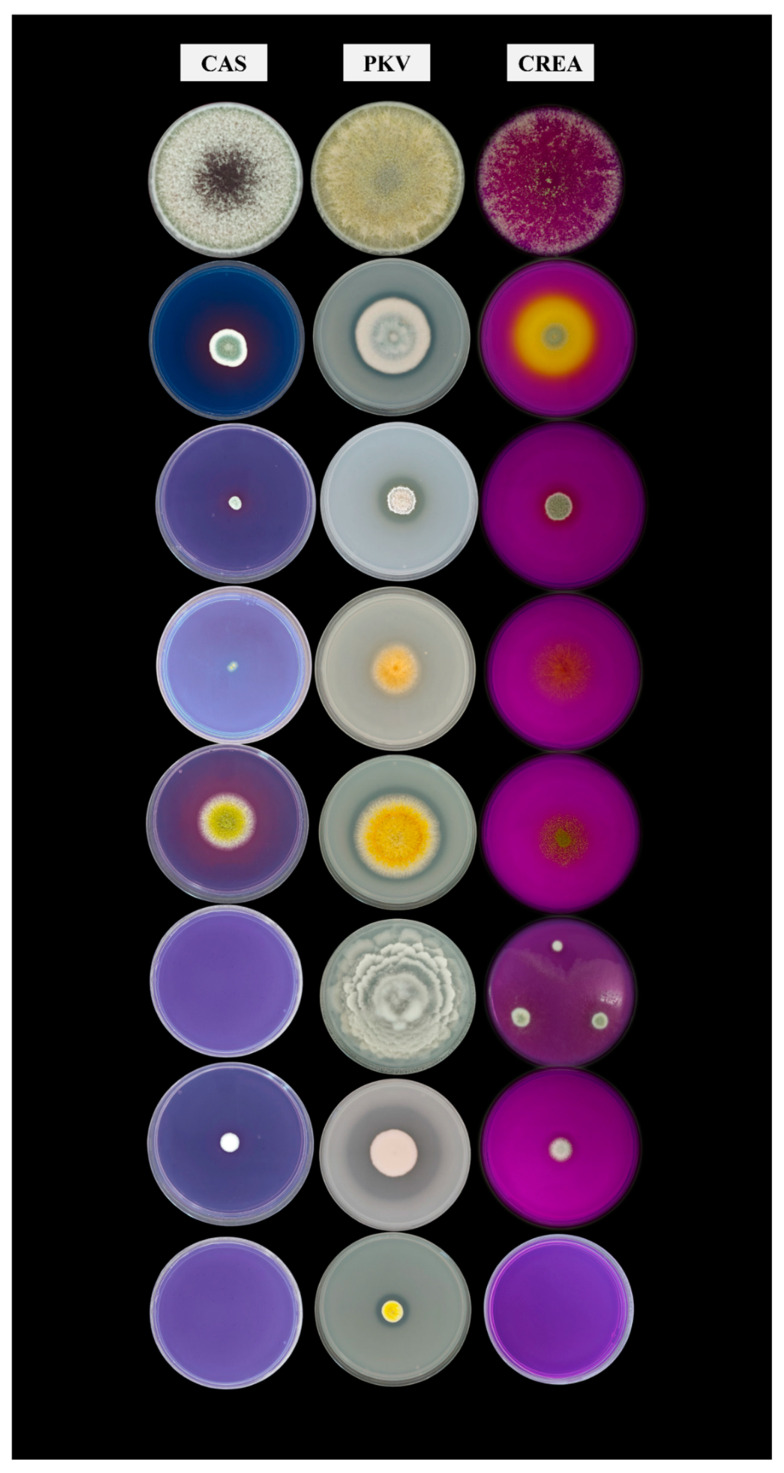
Results of the fungal traits tests. From the left to the right: CAS, PKV, and CREA media plates. From top to bottom, the following strains are *T. harzianum*, *P. piscarium*, *P. glandicola*, *A. luteoalbus*, *A. flavus*, *M. elongata*, *P. chalabudae*, and *Penicillium* sp.

These strains, selected for use as bioinoculants in rice cultivation, were analyzed for their metabolic characteristics. The strains demonstrated an overall positive response in the test, except for the CREA test. All the tested fungi developed on this medium, though without eliciting a positive reaction (Table 1, Figure 1). Strain 022-I8 was the only strain to react, thereby confirming its capacity to produce organic acids as byproducts of sucrose metabolism. In comparison, the other fungi—excluding one native strain (*M. elongata*) and two allochthonous strains (*A. luteoalbus* and *T. harzianum*)—demonstrated the ability to produce organic acids involved in the metabolic reaction of phosphate solubilization, as evidenced by the results obtained on the PKV plates. Furthermore, 62.5% of the tested strains exhibited a positive response in the CAS test, thereby demonstrating a good capacity to produce siderophores.

A comparison among strains was made by calculating the ratio of the halo to colony diameter for the CAS and PKV tests, and the best-performing strains were identified as fungi of the genus *Penicillium*. The computed values ranged from 1.12 to 2.42 (Table 1). In particular, the fungi that demonstrated the most remarkable properties were *P. glandicola* (H/C _CAS_ = 2.33; H/C _PKV_ = 1.67), *P. chalabudae* (H/C _CAS_ = 1.69; H/C _PKV_ = 2.00), and *P. piscarium* (H/C _CAS_ = 2.42, H/C _PKV_ = 1.12). The fungus *Aspergillus flavus* also yielded promising results, exhibiting an H/C _CAS_ of 1.77 and an H/C _PKV_ of 1.13. This qualitative result was consistent with the findings of other studies [51,59].

### 3.2. Secondary Metabolites Production

**Figure 2:**
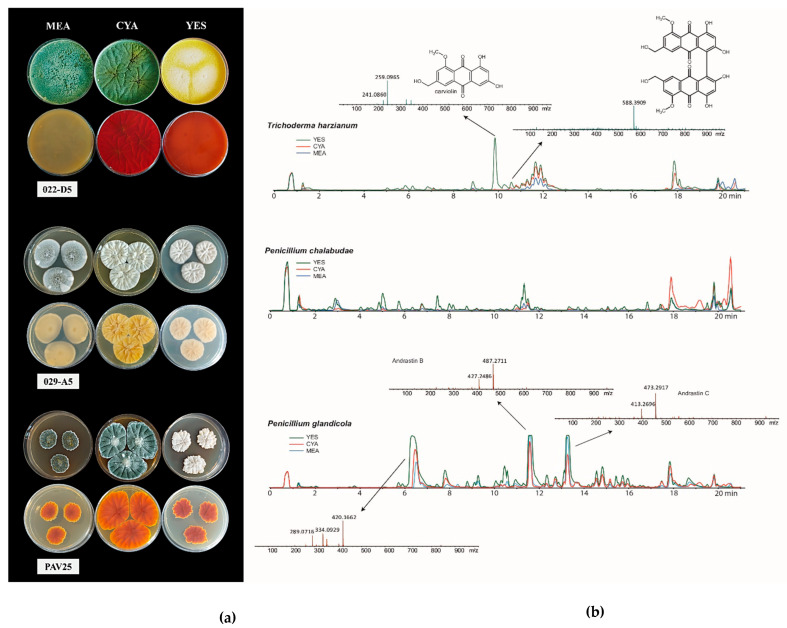
(**a**) Recto and verso of *T. harzianum*, *P. chalabudae*, and *P. glandicola* strains on MEA, CYA, and YES culture media (from top to bottom). (**b**) HPLC-HRMS chromatogram of metabolites detected by analysis.

The analysis of fungal metabolite extracts was conducted by HPLC-HRMS. The target fungi were *T. harzianum*, *P. chalabudae*, and *P. glandicola* (Figure 2). All three fungi produced an extensive array of secondary metabolites. Some of these were tentatively identified as polyketides. *Trichoderma harzianum* (022-D5) produced a considerable amount of a compound tentatively identified as carviolin, a deep red terpene organic compound. For *Penicillium glandicola* (PAV25), we tentatively identified two compounds as andrastin B and andrastin C, based on their MS spectra.

Finally, a peak of a secondary metabolite ([M+H]^+^: 420.1662) was observed in *Penicillium chalabudae* strain (029-A5), for which further analyses will be necessary to determine whether it is indeed a metabolite not yet described in the literature.

### 3.3. Greenhouse Experiment

The eight fungi selected for the study were then cultivated and prepared for the formulation of two separate inocula: one containing the four indigenous strains from the soil used in the experiment and the other containing the fungi isolated from soils from other rice fields. The fungal concentration within each of the two final inocula was 10^7^ CFU/mL, in line with other related studies [60,61]. Tests for fungal viability conducted in the fourth week and the twelfth week revealed the presence of the strains utilized in the two distinct bioinoculants. Specifically, the composition of the fungal community in soil inoculated with native strains was characterized by the prominent presence of the inoculated strains, setting it apart from the fungal community composition observed in the controls.

Three Italian varieties of *Oryza sativa* L. ssp. *japonica* plants were utilized in the study. Cripto and Titanio belong to the group of temperate *japonica* varieties that have been demonstrated to possess a natural hyperaccumulation of arsenic in their grains. Plus is a tropical *japonica* variety that is innately predisposed to cadmium accumulation in grains [62]. All plants demonstrated successful growth and development in every combination of variety, treatment, and growing regime.

**Figure 3:**
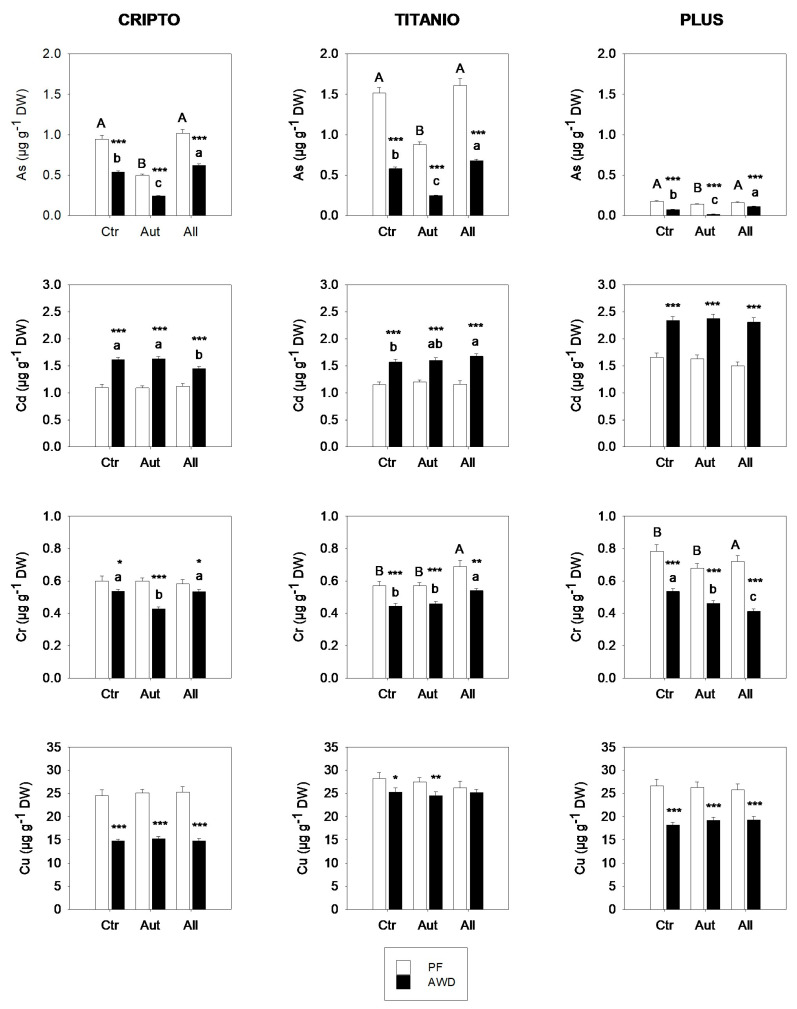
Accumulation of As, Cd, Cr, and Cu in the shoots of three rice varieties grown in soils not inoculated (Ctr) or inoculated with autochthonous (Aut) or allochthonous (All) fungal strains under permanent flooding (PF; white bars) and alternate wetting and drying (AWD; black bars) water regimes. Data are means ± standard deviation from two experiments conducted in quadruplicate. Different letters indicate significant differences among rice varieties (*p* < 0.05). Asterisks indicate significant differences between PF and AWD (Student’s *t*-test; * *p* ≤ 0.05, ** *p* ≤ 0.01, *** *p* ≤ 0.001). DW, dry weight.

The accumulation of arsenic (As), cadmium (Cd), chromium (Cr), and copper (Cu) in the aboveground biomass of rice varied significantly among cultivars, water regimes, and fungal inoculation treatments (Figure 3). As expected, AWD led to consistent declines in As across all treatments and cultivars, with reductions ranging from 31% to 86% relative to the PF. Inoculation with autochthonous fungi significantly reduced arsenic (As) concentrations in rice shoots compared to the control, with the most pronounced reductions observed under alternate AWD conditions. Under AWD, the As content decreased by up to 75% in the Plus cultivar and by over 55% in both Cripto and Titanio. In contrast, inoculation with allochthonous fungi had significant effects only under AWD, where it led to an increase in As accumulation in rice shoots.

In contrast to the trend observed for arsenic, the AWD regime, compared to PF, promoted Cd accumulation in rice shoots across all combinations of fungal inoculum and rice variety. Interestingly, under PF conditions, Cd accumulation was unaffected by fungal inoculation, while under AWD, the effect of the fungi was limited and varied depending on the rice cultivar.

Concerning Cr and Cu, a significant effect of the water regime on the accumulation of these metals in rice shoots was observed, with higher concentrations under permanent flooding (PF) compared to alternate wetting and drying (AWD). These effects were likely related to differences in the soil redox potential induced by the water regime, which influenced the bioavailability of Cr and Cu. The chromium accumulation in rice shoots showed limited variation across treatments, fluctuating by 10–30%. Although some statistically significant differences were observed, no consistent patterns emerged, suggesting that Cr accumulation resulted from complex metal- and cultivar-specific dynamics rather than uniform responses to fungal inoculation. Finally, Cu accumulation was not affected by fungal inoculation in any of the rice varieties analyzed.

## 4. Discussion

The soil in this study was obtained from a paddy field, where elevated concentrations of heavy metals were detected, particularly arsenic (24.91 mg kg^−1^), cadmium (0.94 mg kg^−1^), chromium (114.97 mg kg^−1^), and copper (96.86 mg kg^−1^) (Table A1).

The fungal strains selected and used in this experiment exhibited plant growth-promoting (PGP) properties. Among them, strains belonging to the genera *Penicillium* and *Aspergillus* proved to be the most effective, in line with findings reported in the literature [48,63]. Indeed, these microfungi can interact with the roots of crop plants, thereby enhancing growth [20,60,64,65]. Metabolic characterization was also extended to the analysis of secondary metabolite production. In this study, molecules with unique features and potential applications in various fields were identified as polyketides. In particular, carviolin—a pigment with moderate immunosuppressive activity [66,67]—was detected and is known to be produced by *Penicillium roseopurpureum* [68,69]. In addition, two compounds were tentatively identified as andrastins, polyketides with therapeutic applications due to their potential anticancer activity [70]. One strain, isolated from contaminated soil, produced a metabolite that did not directly match any compound currently described in the literature. As such, further investigations will be conducted. Fungi from extreme environments often represent a rich reservoir of novel bioactive molecules with biotechnological relevance [71,72].

The selected strains were then applied as two bioinoculants in a simulated cultivation system involving three rice varieties grown under two distinct water regimes and in highly contaminated soil. The concentrations of As and Cd in plants exhibited significant variation in response to the water regimes of AWD and PF. In accordance with the literature, the aerobic conditions of the AWD regime resulted in a reduction in the bioavailability of arsenic in soil—present more as AsV, less soluble in water—concurrently increasing the bioavailability of cadmium [2,8,73].

Notably, the use of native strains as bioinoculants has demonstrated efficacy in reducing As concentrations in plant tissues, thereby minimizing its potential impact on both plant and human health. However, at this stage, we are not yet able to fully interpret the specific effects of the bioinoculants on the accumulation of metals and metalloids in rice plants. Nonetheless, it is plausible to speculate that these effects may be linked to the activity of the fungi in the rhizosphere. In particular, the fungi may directly absorb certain metals or metalloids, thereby reducing their availability for root uptake [74]. Additionally, they may alter the chemical environment of the rhizosphere—through processes such as pH modification, organic acid secretion, or metal chelation—thus influencing the mobility and bioavailability of these elements [75,76,77]. Alternatively, the fungi might induce physiological changes in the host plants that affect the metal uptake pathways and the mechanisms involved in their transport and sequestration [78,79]. Interestingly, the variety-specific patterns observed in Cd accumulation under the AWD regime suggest the existence of complex interactions between the fungal inoculants and the root systems of different rice genotypes, potentially influencing metal uptake dynamics in a genotype-dependent manner. The use of PGP organisms in plant cultivation systems has been demonstrated to improve nutrient availability and mitigate stress factors [38,80,81]. This approach can limit the mobility of metals within the soil–plant system and their potential entry into food webs [23,82,83].

## 5. Conclusions

The isolation of fungal strains from paddy field soils, followed by their metabolic characterization, enabled the selection of the most promising plant growth-promoting fungi (PGPF). The application of indigenous soil fungi, naturally adapted to high levels of metal contamination, significantly reduced arsenic accumulation across all rice varieties. These findings support the use of autochthonous fungi in nature-based solutions (NBS) to improve crop performance and soil quality in environmentally challenging conditions.

The proposed approach aims to develop a methodology capable of addressing the needs of growers cultivating crops in contaminated or otherwise adverse environments. The optimization of environmentally sustainable intervention strategies relies on the customization of bioinoculants according to the specific characteristics of the target soil. This strategy, due to its adaptability, has the potential to be applied to a wide range of crops beyond rice. Future studies should focus on combining biotechnological and agronomic strategies with local environmental conditions to maximize the efficacy of this approach.

## Figures and Tables

**Table 1:** Data on the colony development and diameters (C) and reaction halos (H) of the fungi assayed on CAS and PKV. The ratio between H and C was calculated.

**Fungus**	**Growth on CAS**	**Halo Diameter (cm)**	**Colony Diameter (cm)**	**Ratio H/C**
**Native fungi**				
*Aspergillus flavus*	+	6.43	3.63	1.77
*Mortierella elongata*	-	0.00	0.00	/
*Penicillium* sp.	-	0.00	0.00	/
*Penicillium chalabudae*	+	1.78	1.05	1.69
**Allochthonous fungi**				
*Acrostalagmus luteoalbus*	+	0.00	0.00	/
*Penicillium glandicola*	+	1.75	0.75	2.33
*Penicillium piscarium*	+	6.37	2.63	2.42
*Trichoderma harzianum*	+	9.00	9.00	1.00
				
**Fungus**	**Growth on PKV**	**Halo diameter (cm)**	**Colony diameter (cm)**	**Ratio H/C**
**Native fungi**				
*Aspergillus flavus*	+	6.18	5.45	1.13
*Mortierella elongata*	+	0	7.78	0.00
*Penicillium* sp.	+	1.9	1.3	1.46
*Penicillium chalabudae*	+	3.75	1.88	2.00
**Allochthonous fungi**				
*Acrostalagmus luteoalbus*	+	0	3.33	0.00
*Penicillium glandicola*	+	2.67	1.6	1.67
*Penicillium piscarium*	+	4.87	4.33	1.12
*Trichoderma harzianum*	+	0	9	0.00

## Data Availability

For any questions about data, contact the corresponding author.

## Abbreviations

The following abbreviations are used in this manuscript:

NBS	Nature-based solutions
PF	Permanent flooding
AWD	Alternate wetting and drying
PGPF	Plant growth-promoting fungi
PGPB	Plant growth-promoting bacteria

## Appendix A

**Table A1:** Physico-chemical properties of the experimental soil. CEC: cation exchange capacity; TOC: total organic carbon; SOM: soil organic matter; TKN: total Kjeldahl nitrogen; C/N: carbon-to-nitrogen ratio; available P_2_O_5_ (extracted with the Olsen method).

Parameter	Value	Confidential Interval
**pH**	6.51	0.19
**Sand (% DM)**	55	5
**Silt (% DM)**	36	1
**Clay (% DM)**	9	2
**Soil texture classification**	Sandy loam	
**CEC (cmol(+) kg^−1^)**	18.85	0.95
**TOC (g kg^−1^)**	14.7	1.1
**SOM (g kg^−1^)**	25.4	1.9
**TKN (g kg^−1^)**	1.78	0.17
**C/N**	8.3	0.2
**Available P_2_O_5_ (mg kg^−1^)**	178	9
**Total Arsenic (As) (mg kg^−1^)**	24.91	1.87
**Cadmium (Cd) (mg kg^−1^)**	0.94	0.05
**Copper (Cu) (mg kg^−1^)**	96.86	3.55
**Lead (Pb) (mg kg^−1^)**	49.51	2.52
**Mercury (Hg) (mg kg^−1^)**	<0.1	
**Nickel (Ni) (mg kg^−1^)**	98.19	4.43
**Selenium (Se) (mg kg^−1^)**	0.61	0.05
**Total Chromium (Cr) (mg kg^−1^)**	114.97	5.84
**Zinc (Zn)**	359.50	9.99
